# CREATING INTERNET-BASED DISTANCE LEARNING IN GERONTOLOGY

**DOI:** 10.1093/geroni/igad104.0797

**Published:** 2023-12-21

**Authors:** Edward Schneider

**Affiliations:** University of Southern California, Los Angeles, California, United States

## Abstract

Dr. Schneider was Associate Director and then Deputy Director of the National Institute on Aging from 1980 through 1986. Dr. Schneider helped to create the Geriatric Leadership Academic Award. This award funded geriatric positions in many medical schools including Harvard, Johns Hopkins, Duke the University of California at Los Angeles, and the University of Tennessee. These awards led the creation of Geriatric Divisions at numerous American medical schools. After he became Dean of the Leonard Davis School of Gerontology, Dr. Edward Schneider with Maria Henke created the nation’s first Internet based Master’s Degree program in Gerontology. This program has graduated over 1000 students since then.

